# The Ratio of Linoleic and Linolenic Acid in the Pre-hibernation Diet Influences NFκB Signaling in Garden Dormice During Torpor

**DOI:** 10.3389/fmolb.2020.00097

**Published:** 2020-05-21

**Authors:** Samantha M. Logan, Alexander J. Watts, Annika Posautz, Anna Kübber-Heiss, Johanna Painer, Gabrielle Stalder, Sylvain Giroud, Kenneth B. Storey

**Affiliations:** ^1^Department of Biology, Carleton University, Ottawa, ON, Canada; ^2^Department of Interdisciplinary Life Sciences, Research Institute of Wildlife Ecology, University of Veterinary Medicine, Vienna, Austria

**Keywords:** garden dormouse, hibernation, linoleic acid, linolenic acid, inflammation, apoptosis, antioxidant

## Abstract

The fatty acid composition of a pre-hibernation diet can influence the depth and duration of metabolic suppression achieved by hibernators. More specifically, a diet high in *n*−6 polyunsaturated fatty acids (PUFAs) relative to *n*−3 PUFAs is essential to maximize torpor expression. However, few studies have investigated how diets with different *n*−6/*n*−3 PUFA ratios change stress-inducible cell signaling. Garden dormice (*Eliomys quercinus*) were fed one of three diets designed with different ratios of *n*−6 PUFA linoleic acid (LA) and *n*−3 PUFA linolenic acid (ALA). Then, NFκB signaling was assessed in the white adipose, brown adipose, and liver tissues of euthermic and hibernating dormice via multiplex and RT-qPCR analyses of relative protein and transcript levels, respectively. Dormice fed a high LA diet regulated NFκB signaling in a protective manner in all tissues. NFκB signaling was generally decreased in the high LA group, with significant decreases in the protein levels of NFκB mediators IKKα/β, IκBα, and downstream pro-apoptotic protein FADD. Liver and white adipose from torpid dormice fed a high LA diet increased *sod2* expression relative to the other diets or relative to euthermic controls, indicating protection against ROS generated from potentially increased β-oxidation of *n*−6 PUFAs. The low LA diet increased biomarkers for apoptosis relative to other diets and relative to euthermia, suggesting low LA diets may be detrimental to hibernator health. Overall, this study suggests that changes in the ratio of *n*−6/ *n*−3 PUFAs in the diet influences apoptotic and antioxidant responses in white adipose, brown adipose, and liver of hibernating garden dormice.

## Introduction

To feed a garden (dormouse) is to feed our mind and our soul. This take on an old proverb highlights our species' fascination with natural history, where understanding the impact of changes in diet (e.g., through changing the available foods of wild species by planting different crops) is not only important for species conservation, but can also help us understand the limits of our own metabolism for academic and medicinal breakthroughs. Herein, we explore how changes in diet affect the metabolism of one of the world's most adaptable mammalian species, the garden dormouse (*Eliomys quercinus*). For decades, scientists have changed the proportions, total amounts, and quality of carbohydrates, proteins, and triglycerides in the human and often, homeothermic rodent diet, in order to better understand everything from energy expenditure (fuel usage, thermogenesis) and disease (e.g., obesity, diabetes, heart disease, etc.), to the importance of the microbiome. How do nature's most extreme, and most adaptable species handle changes in diet? To explore this idea, we focus on the metabolic effects of changes in the diets of garden dormice, a small mammalian hibernator. Hibernators are a special kind of heterothermic mammal that can survive long periods with little to no available food and/or periods of intense cold using an adaptation called metabolic rate depression, also known as torpor. Metabolic reduction involves slowing all but the most important cellular processes (control of cell death, antioxidant defenses, fatty acid metabolism, kinase-mediated regulation of cellular signaling, among others), a decline in body temperature (approaching ambient), and a slowing of key physiological measures like brain activity, as well as heart and breathing rates (Frerichs et al., 1994; Wang and Lee, 1996). Hibernators are either food-storing or fat-storing. Fat-storing hibernators switch from carbohydrate metabolism to fat oxidation to sustain their basal metabolic rate and most essential physiological processes. To sustain 6–9 months of torpor (depending on the species), fat-storing hibernators must become hyperphagic in the fall before winter hibernation, such that they gain massive amounts of fat stores to use as the main energy source during torpor. How diet impacts metabolism in species capable of torpor would be of great interest, for its importance in understanding how these adaptable animals would be able to survive severe climate change or a change in land use, resulting in a transformation of available food types.

The garden dormouse is a fat-storing hibernator that resides in the gardened, rocky, and treed areas of Europe (Perez et al., 2013). During autumn, the garden dormouse typically feasts on insects—specifically orthoptera and coleoptera, in Spain (Gil-Delgado et al., 2010). Most other hibernator species similarly show a need for high levels of fatty acids and protein in their pre-hibernation diets, which also consist of insects and/or seeds (Frank and Storey, 1995, 1996; Frank et al., 2008; Levin et al., 2013). Pre-hibernation diets have high levels of polyunsaturated fatty acids (PUFAs), a nutrient which hibernators consume in their diets that can influence hibernation cycle duration and depth. More specifically, it is the ratio between *n*−6 and *n*−3 PUFAs that is responsible for the observed effects on torpor bout length and body temperature (Ruf and Arnold, 2008). Ground squirrels and marmots fed a diet with a greater amount of *n*−3 PUFAs tend to spend less time in torpor if they enter torpor at all, while hibernators fed diets higher in *n*−6 PUFAs have more ample WAT stores, lose less mass over the hibernation period, and have larger reductions in body temperature during torpor (Frank and Storey, 1995; Hill and Florant, 2000). Further, feeding dormice diets with differing ratios of *n*−6 and *n*−3 PUFAs has been shown to alter WAT fatty acid composition before and during torpor (Giroud et al., 2018).

For mammals, PUFAs are essential nutrients and include “anti-inflammatory” omega-3 fatty acids such as linolenic acid (ALA, 18:3 *n*−3, octadecatrienoic acid) and the overabundant pro-inflammatory omega-6 fatty acids like linoleic acid (LA, 18:2 *n*−6). LA is proposed to influence inflammatory molecular pathways through its ability to induce arachidonic acid (AA) accumulation, which leads to the synthesis of pro-inflammatory eicosanoids and prevents the synthesis of eicosanoids that are later converted to anti-inflammatory products (Schmitz and Ecker, 2008). However, the literature does not support an increase in AA with an increase in LA in the diet, nor does it confirm that inflammatory markers increase in the blood following diets high in LA (Rett and Whelan, 2011; Johnson and Fritsche, 2012; Innes and Calder, 2018). ALA is an anti-inflammatory PUFA that is readily converted to docosahexaenoic acid (DHA) and eicosapentaenoic acid (EPA). Since both fatty acids are converted to downstream products by the same enzymes, it is the ratio of *n*−6/*n*−3 fatty acids that is important to consider when analyzing the relationship between diet composition and downstream metabolism (Schmitz and Ecker, 2008; Innes and Calder, 2018).

ALA and LA are implicated in the regulation of inflammation via intricate links to the nuclear factor kappa B (NFκB) pathway, a transcription factor-mediated signaling pathway that can lead to an increase in inflammatory protein levels. Phosphorylated IKKα/β can phosphorylate the inhibitor IκB, which results in IκB's release from NFκB and its degradation, ultimately leading to the activation of NFκB. Briefly, diets high in LA differentially regulate IKKα/β phosphorylation and NFκB activation, and oppositely, diets low in LA (omega-6) or high in ALA (omega-3) have the potential to inhibit NFκB activity, including the expression of genes such as c-Myc (Poletto et al., 2015; Jangale et al., 2016). ALA inhibits NFκB through the activation of PPARγ and the alteration of cell differentiation pathways (Nakanishi and Tsukamoto, 2015). Since hibernators favor diets rich in *n*−6 PUFAs in the months leading up to winter hibernation, and even incorporate more *n*−6 PUFAs into their cell membranes in advance of hibernation, they may have enhanced NFκB signaling during torpor. Activated NFκB can also trigger the expression of genes involved regulating cell death, cell cycle, and the antioxidant response, such as *myc, sod2, bclxl*, and *bax* (La Rosa et al., 1994; Grimm et al., 2005; Chen et al., 2013). For this reason, we have decided to study the influence of ALA and LA diets on NFκB activation during natural mammalian torpor in the garden dormouse.

## Methods

### Animals

Twenty-five garden dormice (*Eliomys quercinus*), weighing 132.8 ± 3.5 g [100–170 g CI] at pre-hibernation, issued from a breeding colony kept at the Research Institute of Wildlife Ecology (Vienna, Austria), were used in these experiments. During their pre-hibernation fattening (Sept. 2015 & Sept. 2016), animals were housed singly in cages (60 × 40 × 40 cm), each equipped with one nest, bedding and nesting material, and were kept under natural fluctuations of ambient temperature and photoperiod. During the subsequent hibernation period (Oct. 2015/2016 to Jan. 2016/2017), dormice were housed individually in standard laboratory cages (36 × 20 × 14 cm), each provided with a customized nest and bedding material, and kept at 4°C in ventilated cooling units (refrigerators; Liebherr GKv 5730) under constant darkness, without food and water.

### Ethics Statement

All procedures were approved by the institutional ethics committee and the national Austrian authority in accordance to the Austrian Animal Experimentation Act, “Tierversuchsgesetz 2012” (BMBWF-68.205/0137-WF/V/3b/2014).

### Protocol Overview

During the pre-hibernation fattening period, dormice were fed chow enriched with specific oils (see below for more details). Specifically, 8 animals were randomly assigned to each diet group, with 9 animals being fed the high LA diet. Prior to hibernation experiments, the animals were implanted with small temperature transmitters and core body temperature was monitored via a telemetry system (see below for further details). Once animals were spontaneously entering prolonged (>24 h) torpor, hibernation was induced by housing the animals at 4°C without food and water. Hibernation was monitored during the next 3 months until animals were sacrificed at mid-winter (Dec-Jan 2015/2016–2016/2017). Animals were sacrificed either in torpor or during interbout euthermia, at a body temperature of 4.61 ± 0.27°C or 37.13 ± 0.10°C, respectively, by immediate decapitation (if torpid) or by CO_2_-euthanasia then decapitation (when euthermic). These experiments resulted in *n* = 4 animals fed one of the three diets and sampled in torpor or interbout arousal, with the exception of the high LA diet condition where *n* = 5 dormice were sampled during torpor. Tissues were quickly sampled and immediately flash frozen in liquid nitrogen (−196°C) and stored at −80°C until shipped to Carleton University on dry ice.

### Diets

During the fattening phase, dormice were fed one of the three specific diets, each differing in its lipid composition. These diets were made by adding a 10-wt % of linseed oil as the source of *n*−3 fatty acids (notably ALA 18:3 *n*−3), or safflower oil as the source of *n*−6 fatty acids (mainly LA 18:2 *n*−6), or a 5-wt % of linseed oil and 5-wt % of safflower oil to the pellets with which animals are routinely fed in the colony (Topix, Saturn Petcare GmbH, Bremen, Germany). Details of the exact composition of the colony diet is available in Mahlert et al. (2018) (Mahlert et al., 2018). This led to either low, intermediate or high amounts of LA, reversely mirrored by the contents of ALA, in the diets that were termed low LA (“LOW”), intermediate LA (“INT”), and high LA (“HIGH”), respectively. The fatty acid compositions of the diets are summarized in Table 1. Pellets were kept in sealed bags filled with nitrogen at −80°C to minimize peroxidation until use. Fresh pellets were fed to the animals every 2 days, and uneaten food was discarded. During their fattening phase, each group of dormice was fed the experimental diet for at least 14 days, which has been shown by previous studies on small rodents to be sufficient to ensure maximum changes in the fatty acid composition of membranes and tissues (Swanson and Kinsella, 1986; Swanson et al., 1987). To ensure that the fatty acid composition of tissues of the animals differs significantly between the three dietary groups, we determined the fatty acid composition of the white adipose tissue (WAT) from the animals at mid-hibernation (Table 2).

**Table 1:** Fatty acid composition of linseed oil- (“LOW”), safflower oil- (“HIGH”), and linseed/safflower oil (“INT”) -enriched diets as fed to garden dormice for at least 2 weeks during their pre-hibernation fattening phase.

**Fatty acid**	**LOW**	**INT**	**HIGH**
C14:0	0.62	0.62	0.61
C15:0	0.08	0.08	0.08
C16:0	13.55	13.95	14.29
C16:1 (*n*−7)	1.55	1.60	1.60
C17:0	0.18	0.18	0.16
C18:0	6.26	5.64	5.00
C18:1 (*n*−9)	25.94	24.61	23.25
C18:2 (*n*−6)	19.28	35.55	52.95
C18:3 (*n*−3)	31.92	17.16	1.35
C20:4 (*n*−6)	0.34	0.33	0.35
C20:5 (*n*−3)	0.05	0.09	0.09
C22:5 (*n*−3)	0.08	0.06	0.09
C22:6 (*n*−3)	0.15	0.15	0.18
PUFA	51.82	53.34	55.01
MUFA	27.49	26.20	24.86
SFA	20.69	20.46	20.14
∑*n*−6	19.62	35.87	53.29
∑*n*−3	32.20	17.47	1.71
*n*−6/*n*−3	0.61	2.06	31.50
LA/ALA	0.61	2.08	41.22

*Fatty acid proportions are expressed as % of total fatty acids. Values are means from four analyses per diet. “PUFA” refers to polyunsaturated fatty acids, “MUFA” to monounsaturated fatty acids, “SFA” to saturated fatty acids, “∑n−6” to the sum of n−6 PUFA, ∑n−3 to the sum of n−3 PUFA, “n−6/n−3” to the ratio between the sum of n−6 PUFA and the sum of n−3 PUFA, and “LA/ALA” to the ratio between linoleic acid (LA 18:2 n−6) and linolenic acid (ALA 18:3 n−3)*.

**Table 2:** Fatty acid proportions (% of total fatty acids), at mid-hibernation, of total lipids from white adipose tissue (means ± standard deviation) and ratios of certain fatty acid proportions of garden dormice fed diets enriched with either *n*−3 fatty acids (“LOW”) or *n*−6 fatty acids (“HIGH”) or a mix of *n*−3 and *n*−6 fatty acids (“INT”).

**Fatty acid**	**LOW**	**INT**	**HIGH**	**ANOVA**	
				**F-statistic**	***p*-value**
C14:0	1.45 ± 0.05	1.56 ± 0.06	1.64 ± 0.05	3.38	0.07
C15:0	0.12 ± 0.08	0.09 ± 0.04	0.01 ± 0.01	3.33	0.30
C16:0	8.71 ± 0.47^a^	10.19 ± 0.37^b^	10.56 ± 0.24^b^	7.26	*<0.01*
C16:1 (*n*−7)	4.46 ± 0.24	4.60 ± 0.27	3.95 ± 0.21	2.09	0.16
C17:0	0.21 ± 0.09	0.09 ± 0.02	0.03 ± 0.02	3.06	0.08
C18:0	2.19 ± 0.07	2.34 ± 0.09	2.38 ± 0.16	0.72	0.50
C18:1 (*n*−9)	41.11 ± 0.94^a^	45.08 ± 0.53^b^	50.33 ± 0.91^c^	31.36	*<0.001*
C18:2 (*n*−6)	15.87 ± 0.76^a^	26.35 ± 0.62^b^	39.45 ± 0.84^c^	251.80	*<0.001*
C18:3 (*n*−3)	16.28 ± 0.78^a^	9.28 ± 0.35^b^	0.53 ± 0.05^c^	284.20	*<0.001*
C20:4 (*n*−6)	0.20 ± 0.02	0.22 ± 0.02	0.29 ± 0.03	3.38	0.07
C20:5 (*n*−3)	0.06 ± 0.02^ab^	0.07 ± 0.01^a^	0.02 ± 0.01^b^	4.59	*<0.05*
C22:5 (*n*−3)	0.07 ± 0.01^a^	0.08 ± 0.01^a^	0.02 ± 0.01^b^	5.92	*<0.05*
C22:6 (*n*−3)	0.06 ± 0.02	0.07 ± 0.02	0.01 ± 0.01	2.54	0.12
PUFA	32.54 ± 0.54^a^	36.05 ± 0.79^b^	40.33 ± 0.83^c^	28.22	*<0.001*
MUFA	54.79 ± 0.78^a^	49.68 ± 0.72^b^	45.06 ± 1.07^c^	30.37	*<0.001*
SFA	12.68 ± 0.46^a^	14.27 ± 0.40^b^	14.61 ± 0.41^b^	5.89	*<0.05*
∑*n*−6	16.07 ± 0.75^a^	26.57 ± 0.62^b^	39.74 ± 0.84^c^	254.00	*<0.001*
∑*n*−3	16.47 ± 0.78^a^	9.49 ± 0.37^b^	0.59 ± 0.07^c^	284.70	*<0.001*
*n*−6/*n*−3	1.00 ± 0.09^a^	2.83 ± 0.11^a^	74.19 ± 7.28^b^	86.73	*<0.001*
LA/ALA	1.00 ± 0.09^a^	2.87 ± 0.11^a^	77.64 ± 5.74^b^	152.90	*<0.001*

“*PUFA” refers to polyunsaturated fatty acids, “MUFA” to monounsaturated fatty acids, “SFA” to saturated fatty acids, “∑ n−6” to the sum of n−6 PUFA, “∑ n−3” to the sum of n−3 PUFA, “n−6/n−3” to the ratio between the sum of n−6 PUFA and the sum of n−3 PUFA, and “LA/ALA” to the ratio between linoleic acid (LA 18:2 n−6) and linolenic acid (ALA 18:3 n−3). Where it applies, dietary groups differing significantly with p < 0.05 (Tukey–like post-hoc comparisons) are denoted by different superscripts. Significant p-values are shown in italic*.

### Surgical Implantations of Transmitters and Body Temperature Measurements

Transmitters (model: TA-10TA-F10, 1.1cc, 1.6g, accuracy: 0.15°C; Data Sciences International, St Paul, US) were calibrated prior to implantation between 0 and 40°C in a temperature-controlled water bath. Transmitters were surgically implanted under anesthesia induced by subcutaneous injection of 50 mg kg^−1^ ketamine (Ketamidor® 10%, Richter Pharma, Wels, Austria) and 5 mg kg^−1^ xylazine (Rompun® 2%, Bayer, Leverkusen, Germany), as previously reported (Giroud et al., 2018). Animals were then maintained with 1.5% isoflurane in an oxygen stream via facemask. For post-operative analgesia 5 mg kg^−1^ ketoprofen (Romefen 10% Merial S.A.S., Toulouse, France) was administered subcutaneously. The operation field was prepared according to standard surgical procedures and covered by sterile surgical drapes. Animals were placed in dorsal recumbency and the abdominal cavity was opened through a 1 cm incision in the *linea alba* to introduce the temperature transmitter within the abdomen. Peritoneum and abdominal muscles were sutured using synthetic absorbable surgical suture material USP 3/0 (Surgicryl PGA, SMI AG, Hünningen, Belgium) using a single-button suture technique. In addition, synthetic absorbable surgical suture material USP 4/0 (Surgicryl PGA, SMI AG, Hünningen, Belgium) was used to suture the skin with an intra-cutaneous suture technique. During the entire procedure, vital parameters [respiration rate, peripheral hemoglobin oxygen saturation as measured by pulse oximetry (SpO_2_), and heart rate] were monitored. After surgery, all animals recovered for a period of 10 days before starting temperature recordings. A receiver board (RPC-1; Data Sciences International, St Paul, US) was positioned under each individual cage to collect the radio frequency signals from transmitters. Body temperature was recorded for 10 s every 5 min. Data were analyzed using the Dataquest software (LabPro Data Sciences).

### Lipid Analysis

Lipid analysis was performed as previously described in garden dormice (Giroud et al., 2018). Briefly, total lipids were extracted from both diets and WAT following the procedure of Folch et al. (1957). Since triglyceride fatty acids represent >95% of total lipids in rodent WAT (Florant et al., 1990), triglycerides and PLs in WAT were not separated prior to analysis. Samples were trans-esterified with a one-step method (Lepage and Roy, 1986; Eder, 1995). Fatty acid methyl esters (FAME) were identified by gas-liquid chromatography using a Perkin-Elmer FID AutoSystem XL autosampler chromatograph equipped with a 30 m × 0.25 mm × 0.25 μm HP INNOWax capillary column, using the following parameters: injector 240°C, column 130–180°C at 4°C/min, 180–200°C at 3°C/min, 200–240°C at 15°C/min, 240°C for 8 min. The relative fatty acid composition was quantified using external FAME standards (Supelco) run after every 20 samples and Turbochrom 6.3 software (Perkin Elmer). Concentrations of single fatty acids were calculated as mass % of total identified peaks of 13 fatty acids of chain length 14 to 22.

### Protein Extraction and Multiplex Analysis

Frozen tissue samples of brown adipose tissue (BAT), white adipose tissue (WAT), and liver (LIV) were obtained from euthermic (aroused control) and torpid dormice that were fed either low LA, intermediate LA or high LA diets. Approximately 80–100 mg of tissue sample was weighed before being homogenized 1:4 w:v (WAT) or 1:5 w:v (BAT, LIV) with a glass Dounce homogenizer in ice-cold lysis buffer (1 mM Na3VO4, 10 mM NaF, 10 mM β-glycerophosphate, and 10 μL/mL Sigma protease inhibitor), supplemented with a few crystals of the serine protease inhibitor phenylmethylsulfonyl fluoride (PMSF). The homogenized samples were sonicated for 10 s before being incubated on ice for 30 min with vortexing every 10 min. The samples were centrifuged at 10,000 rpm for 20 min at 4°C. Protein concentration determination of the soluble proteins in the supernatant was performed with the Bio-Rad protein dye reagent concentrate (Bio-Rad, Cat# 500-0006), diluted 1:4 v:v with autoclaved ddH_2_O, as per the manufacturer's instructions.

A multiplex assay (EMD Millipore, Cat# 48-630MAG) for six key NFκB proteins was used as directed by the manufacturer. A 10 min room temperature incubation with Assay buffer (EMD Millipore, Cat# 43-010) was performed and the buffer was decanted. Before every decanting step, the plate was positioned on a magnetic separation block for 1 min. Equal volumes of sonicated beads (diluted 20X in Assay buffer) and total protein lysates were added to each well for a total volume of 50 μL. Importantly, BAT protein samples were standardized with Assay buffer to include 2.4 μg/μL (to include 60 μg of protein per well) and WAT and liver samples were all 0.4 μg/μL (to include 10 μg of protein per well). Assay buffer was used instead of protein lysate for the blank, HeLa Cell Lysate: Lambda Phosphatase (EMD Millipore, Cat# 47-229) was used as the unstimulated control, and HeLa Cell Lysate: TNFα/Calyculin A (EMD Millipore, Cat# 47-230) was used as the stimulated control. The appropriate amount of assay buffer was added to the HeLa lysates, as described in the manufacturer's instructions. Protein lysates and controls were incubated with magnetic beads overnight at 4 °C, in the dark, and on a shaker. The contents of the wells were removed and the plate was washed twice with Assay buffer. Then, 1X Millipore MAP Detection Antibody (EMD Millipore, Cat# 44-630KMG) incubated in the wells for 1 hour at room temperature on a shaker, in the dark. The detection antibody was decanted before 1X Milliplex® MAP Streptavidin-Phycoerythrin (SAPE) (EMD Millipore, Cat# 45-001H) was added to the wells for a 15 min, room temperature incubation on a shaker, in the dark. While retaining the SAPE, Milliplex® MAP Amplification Buffer (EMD Millipore, 43-024A) was added to each well and shaken at room temperature for 15 min, in the dark. The well contents were then removed and the beads were resuspended in 150 μL of Assay buffer before mixing on the plate shaker for 5 min, covered, before taking measurements. A Luminex® 200 instrument and xPonent software (Luminex® Corporation) were used to measure Median fluorescent intensity (MFI).

### RNA-Extraction, cDNA Synthesis, and RT-qPCR Analysis

Briefly, ~50–150 mg of frozen WAT, BAT, and liver tissue was weighed for each biological replicate and then homogenized in 1 mL Trizol (Invitrogen, Cat#15596026) using a glass Dounce homogenizer. Of note, there was only enough tissue sample available of WAT to conduct the RNA-extractions on an *n* = 4 of euthermic and torpid dormice fed a low LA diet, and an *n* = 5 of torpid dormice fed a high LA diet. Then 200 μL of chloroform was mixed with the samples before centrifugation at 10,000 × g for 15 min at 4°C. To the isolated upper aqueous phase containing the RNA fraction, 750 μL of 2-propanol was added to precipitate the RNA. Each sample was centrifuged at 12,000 × g for 15 min at 4°C, washed with 1 mL of 70 % ethanol, and then centrifuged again for 12,000 × g for 5 min. The RNA pellets air dried for 15–30 min at room temperature before resuspending in 30 μL autoclaved RNase-free water. RNA purity was assessed by measuring the absorbance at 260 and 280 nm. RNA integrity was assessed by visualizing 18S and 26S ribosomal bands on a 1% agarose gel with SybrGreen staining.

The RNA was used for cDNA synthesis. Firstly, WAT, BAT, and liver RNA samples were standardized using autoclaved RNase-free water to contain 1 μg, 2 μg, and 2 μg of RNA respectively. The RNA from each sample incubated with 1 μL of Oligo-dT (200 ng/μL) at 65°C for 5 min in a thermocycler. Then, the samples were chilled on ice for 5 min. Reverse transcription using the SuperScript™ II Reverse Transcriptase kit (Invitrogen, Cat# 18064014) converted the RNA into cDNA. To the samples, 4 μL of 5X first-strand buffer (Invitrogen), 2 μL of 0.1 M DTT (Invitrogen), 1 μL of 10 mM dNTPs (BioShop, Cat# NUC001), and 1 μL of MMLV Reverse transcriptase (Invitrogen) were added before a 42°C incubation in the thermocycler for 1 h. Since the garden dormouse (*Eliomys quercinus*) is from the order Sciuromorpha, the annotated genome of the 13-lined ground squirrel (*Ictidomys tridecemlineatus*), another member from the same order, was used to design primers with the Primer-BLAST tool on the National Center for Biotechnology Information (NCBI) website. PPIA was used as the reference gene for RT-qPCR quantifications performed for BAT and liver. The primer pairs were purchased from BioBasic Inc. and were as follows: *ppia* forward, 5′- GCA AGT CCA TCT ACG GGG AG-3′; *ppia* reverse, 5′- CTC AGT CTT GGC AGT GCA GA-3′*; bax* forward, 5′-CCT TTT GCT TCA GGG TTT CAT CC-3′*; bax* reverse, 5′- CTT CAG ACA CTC GCT CAG CTT-3′; *sod2* forward, 5′- CAA TAA GGA GCA GGG ACG CT-3′; *sod2* reverse, 5′- CCA GCA GTG GGA TAA GAC CTG-3′; *bclxl* forward, 5′-TCT CTT TCT CTC TCT TCA GAA CCT-3′; *bclxl* reverse, 5′-CTC ACT GAG TCT GGT CTC TGC-3′. PCR reagents were prepared and RT-qPCR was performed as previously described (Pellissier et al., 2006) using a CFX-96 Real-Time PCR Detection System (Bio-Rad, Hercules, California, USA). To ensure the desired product was amplified, melt-curve analysis was used to determine that only a single peak was present representing a single PCR product, and the amplification curve of a two-fold dilution series of the cDNA was analyzed to determine that the primer pairs were not amplifying primer-dimers, as per MIQE guidelines (Bustin et al., 2009).

### Statistical Analyses

Analyses on fatty acid composition were carried out using R 3.5.1 (R Core Team, 2014). The normality of residuals from statistical models was assessed by inspecting quantile-quantile-plots and histograms. If necessary, response variables were Box-Cox transformed to achieve normality. To test for differences in WAT fatty acid composition between the three dietary groups (LOW, INT, HIGH), linear models with Tuckey-like *post hoc* multiple comparison tests were used. All *p*-values linear models were adjusted for multi-comparisons between fatty acid proportions using False Discovery Rate (Benjamini and Hochberg, 1995).

The ΔΔCt method was used in the analysis of RT-qPCR results (Bustin et al., 2009; Taylor et al., 2019). *ppia* was used as the reference gene for all three tissues because its gene expression did not show any statistically significant differences in animals fed different diets (intermediate LA, low LA, high LA) or sampled at different points of metabolic rate (euthermia, torpor).

To determine the statistical significance of the molecular data, two independent analyses were conducted on both the Median fluorescent intensity (MFI) data collected by the multiplex instrument and ΔΔCt values obtained using RT-qPCR. First, to determine if there were any statistically significant differences between the three diet groups (considering only euthermic or only torpor samples during a given analysis), the MFI or ΔΔCt values were analyzed using a One-way ANOVA with a Tukey *post-hoc* (*n* = 3–4 independent preparations per diet condition, *p* < 0.05). Secondly, for each individual diet, the relative gene and protein expression levels of euthermic and torpid animals were compared using a Student's *t*-test where *p* < 0.05 was deemed statistically significant. RT-qPCR data for WAT was analyzed using a One-Way ANOVA with a Dunnett's *post-hoc* test such that gene expression data was compared between control (*n* = 4, with the exception of *sod2* analysis where *n* = 3 was used) and torpid (*n* = 4) groups fed a low LA diet, and between torpid animals fed either a low LA or a high LA diet (*n* = 5, except *n* = 4 for *sod2* gene expression analysis). All molecular analyses were performed using RBioPlot, a statistical analysis package for R, which also generated the histograms that presented the standardized data as mean ± SEM (Zhang and Storey, 2016). The results of the latter statistical analysis were added to the R-generated histograms as bars across two points with an asterisk (^*^) above it, to denote statistical significance between control and torpid animals.

## Results

Relative levels of NFκB signaling proteins or transcripts downstream of NFκB were assessed in three tissues from hibernating and interbout euthermic dormice that were either fed diets rich in LA but deficient in ALA (called High), intermediate levels of both fatty acids (called Intermediate) or low levels of LA with high levels of ALA (called Low).

### White Adipose Tissue (WAT)

In white adipose tissue (WAT) the only change with respect to diet was a decrease in p-IKKα/β (Ser177/Ser181) in torpid animals fed a high LA diet with respect to the intermediate diet control (**Figure 2**). There were no differences in p-IKKα/β (Ser177/Ser181) in euthermic animals fed different diets, and after performing a Student's *t*-test, it was discovered that this difference was not significant between control and torpid animals (*p* = 0.37). By contrast, when animals were fed a low LA diet, total c-Myc protein levels decreased to 44% of the intermediate diet control level in torpid dormice WAT, and this difference was significant with respect to the euthermic control, where c-Myc levels were 40% of the euthermic levels (*p* = 0.005). Finally, there was a decrease in the relative amount of FAS-associated death domain protein (FADD) (Ser194) in mice fed a high LA diet, when comparing euthermic and torpid dormice. Torpid dormice were shown to have p-FADD (Ser194) levels approaching 45% of the total euthermic p-FADD (Ser194) levels (*p* = 0.025).

Torpid dormice fed a low LA diet had significantly less *sod2* transcripts compared to euthermic dormice fed the same diet (**Figure 3**). Indeed, *sod2* levels decreased to just 34.3% of the euthermic level. *Sod2* levels were also low in hibernating dormice fed the low LA diet relative to hibernating dormice fed the high LA diet (1.8-fold change, *p* < 0.05). There were no significant differences in the relative levels of *bax* or *bclxl* comparing the euthermic low LA group or the torpid high LA group to the torpid low LA group.

### Brown Adipose Tissue (BAT)

Brown adipose from garden dormice displayed few alterations in relative protein levels (**Figure 4**). The only difference in relative protein and/or phosphorylation levels of key NFκB signaling proteins between animals fed different diets was a decrease in the level of p-IκBα (Ser32) in euthermic animals fed a high LA diet. Phosphoprotein levels for p-FADD (Ser194), p-IKKα/β (Ser177/Ser181) or p-IκBα (Ser32) were observable in only n=2 torpid dormice fed a low LA, and so these measures were omitted from **Figure 2**. Using the data from dormice fed an intermediate LA diet and a high LA diet (*n* = 4), there were no significant differences in p-FADD (Ser194), p-IKKα/β (Ser177/Ser181) or p-IκBα (Ser32) phosphoprotein levels between euthermic and torpid animals (data not shown). Furthermore, torpid dormice fed either intermediate or high LA revealed no differences in the relative levels of p-FADD (*p* = 0.93), *p*-IKKα/β (*p* = 0.34) or p-IκBα (*p* = 0.44). The only protein whose levels changed significantly between euthermia and torpor was c-Myc, showing a decrease to 38% of the control levels during torpor in dormice fed a low LA diet.

RT-qPCR analysis revealed an increase in both *bax* and *bclxl* transcript levels in the brown adipose tissue from torpid dormice fed a low LA diet compared to the intermediate LA diet, where *bax* levels increased 4-fold and *bclxl* levels increased 2-fold (**Figure 5**). Interestingly, there were no differences in these transcript levels when comparing diets in euthermic dormice. Within the low LA-treated dormice group, *bclxl* transcript levels were significantly elevated by 2.5-fold in torpor compared to euthermic dormice (*p* = 0.02) but the difference in *bax* levels were not significant between control and torpor (*p* = 0.07).

### Liver

No significant differences in NFκB signaling protein or phosphoprotein levels were observed in liver when comparing diets nor when comparing euthermia to torpor (**Figure 6**).

However, liver from dormice treated with three diets of varying LA and ALA ratios showed an increase in the gene expression of *bax* and *sod2* but not *bclxl* (**Figure 7**). Transcript levels of *bax* increased 1.6-fold during torpor compared to euthermic animals when the animals were fed a low LA diet (*p* = 0.04). Furthermore, when comparing animal groups fed different diets, there was a significant increase in *bax* gene expression in torpid animals fed a low LA diet (to 1.6-fold the intermediate LA level). By contrast, *sod2* levels increased 1.6-fold during torpor relative to the euthermic control in animals fed a high LA diet, but there was no difference in *sod2* levels when comparing multiple diets.

## Discussion

### WAT From Dormice Fed Diets High or Low in LA Have Decreased NFκB Signaling but High LA Diets May Induce the Antioxidant Response

**Figure 1:**
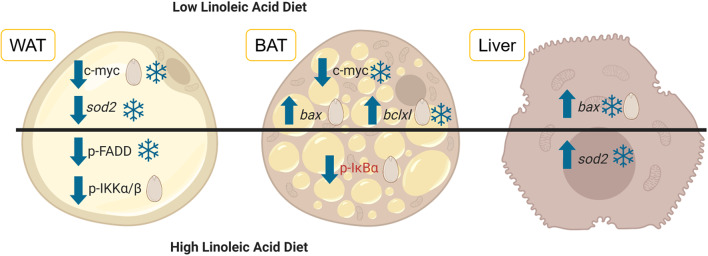
Diets differing in the levels of linoleic acid (LA) and alpha-linolenic acid (ALA) were fed to garden dormice before they entered hibernation naturally. White adipose tissue (WAT, depicted on the left), brown adipose tissue (BAT, shown in the middle) and liver tissue (depicted on the right) were extracted from dormice before and during torpor and the levels of key proteins and genes involved in the regulation of the response to oxidative stress and inflammation were assessed using multiplex antibody-based assays or RT-qPCR. The results of this study are summarized here where arrows indicate whether relative protein levels (normal font) or mRNA (italic font) increased or decreased relative to the euthermic control (snowflake) or relative to the intermediate LA diet (seed). Changes that occurred in torpid animals use blue lettering and the one change that occurred in euthermic animals uses red lettering. The image was created using BioRender. Abbreviations are as follows: p-FADD, phosphorylated Fas Associated Via Death Domain; p-IKKα/β, phosphorylated Inhibitor Of Nuclear Factor Kappa B Kinase subunits alpha and beta; p-IκBα, phosphorylated NF-Kappa-B Inhibitor Alpha; bax, BCL2 Associated X Protein; bclxl, BCL2 Like 1; sod2, Superoxide dismutase 2.

**Figure 2:**
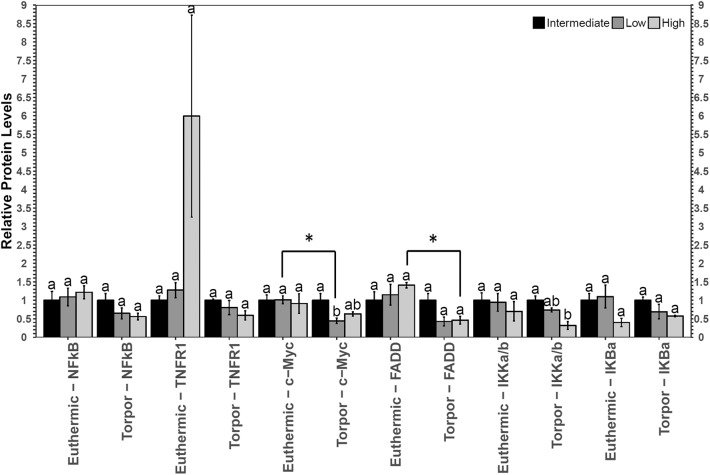
Multiplex analysis of key NFκB proteins in garden dormouse white adipose tissue (WAT). Histogram shows mean relative expression levels of phosphoprotein [p-NFκB (Ser536), p-FADD (Ser194), p-IKKα/β (Ser177/Ser181), and p-IκBα (Ser32)] and total protein (c-myc and TNFR1) (±SEM, *n* = 3–4 independent protein isolations from different animals). Diet data were analyzed using a One-Way ANOVA followed by a Tukey *post-hoc* test, where shared letters represent no significant difference between those diets (*p* > 0.05) and different letters represent significant differences (*p* < 0.05). A Student's *t*-test was used to determine if there were any significant differences in protein levels comparing euthermic controls and hibernated animal groups. Asterisks (*) above the bar that designates the compared points indicates statistical significance with respect to the euthermic control time point.

For the most part, the diets given to the dormice and the hibernating state (torpid vs. euthermic) did not influence pro-apoptotic signaling much in WAT. For an overview of the differentially regulated genes in each of the three tissues, see Figure 1. It was expected that diet might have an influence on the relative levels of tumor necrosis factor receptor 1 (TNFR1) and NFκB phosphorylation, since diets high in LA trigger the phosphorylation of IKKα/β, the inhibition of IκB, and an increase in NFκB transcriptional activation (Poletto et al., 2015). Indeed, Table 2 demonstrates a clear difference between the fatty acid compositions of WAT in dormouse fed each of the three diets, especially with respect to *n*−3 and *n*−6 PUFA levels. Despite significantly different levels of LA and ALA in the WAT of dormice fed the intermediate or low LA diets compared to high LA fed dormice, there were few changes in the protein and phosphorylation patterns that would indicate an increase in NFκB activity. Phosphorylated NFκB (Ser536) levels remained constant in the WAT of dormice from all treatment groups (Figure 2), similar to NFκB expression in the WAT of hibernating 13-lined ground squirrels (Rouble and Storey, 2015). Unexpectedly, during torpor, high LA conditions resulted in a decrease in the phosphoprotein levels of the inhibitor of nuclear factor kappa-B kinase (IKKα/β) at Ser177/Ser181, compared to intermediate LA conditions. When phosphorylated, IKKα/β phosphorylates the NFκB inhibitor, IκB, leading to its inhibition via ubiquitination and degradation. Together with maintained p-IκBα (Ser32) levels, these results suggest that NFκB is likely inhibited in dormice fed high LA diets. In other models, LA and ALA can affect TNFα-mediated NFκB signaling by physically inserting into cell membranes, ultimately changing membrane fluidity and the makeup of the proteins that interact with cytosolic membrane lipid rafts (Schübel et al., 2017). As such, future studies are warranted to investigate the distribution of *n*−3 and *n*−6 PUFAs within the dormouse cell to better explain how LA and ALA may influence the signaling of NFκB and other transcription factors.

**Figure 3:**
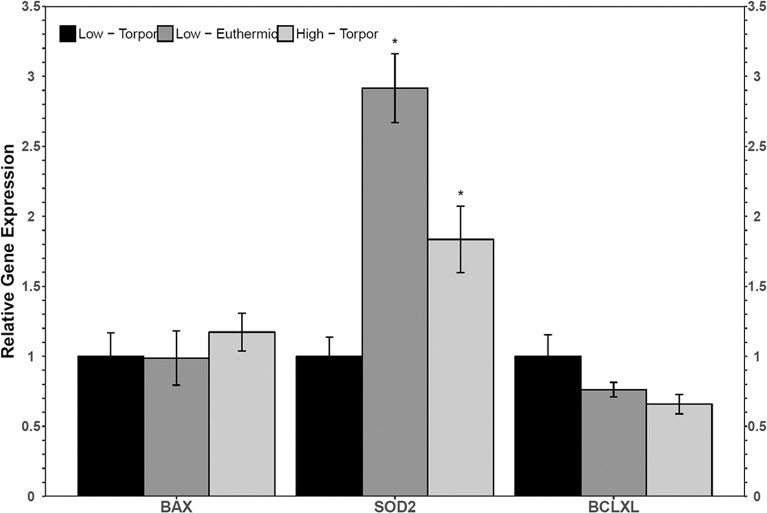
Relative gene expression levels of NFκB target genes, bax, sod2, and bclxl in garden dormouse white adipose tissue (WAT). Histogram shows mean relative cDNA levels following normalization with the reference gene, ppia (±SEM, *n* = 4 independent protein isolations from different animals fed a low LA diet, and *n* = 5 from different animals fed a high LA diet. Exceptions to this were used in sod2 analysis where an *n* = 3 low LA euthermic and *n* = 4 high LA torpor were used). Gene expression data from animals fed a low LA diet sampled during torpor were set to 1 and compared to animals fed a low LA diet and sampled in euthermia, or compared to animals fed a diet high in LA but sampled during torpor. Data were analyzed using a One-Way ANOVA followed by a Dunnett's *post-hoc* test, where asterisks (*) represent statistical significance relative to animals fed a low LA diet and sampled during torpor.

To develop a better understanding of the activation status of NFκB in hibernating dormice fed diets with different ratios of *n*−3 and *n*−6 PUFAs, the relative abundance of NFκB downstream genes were measured using RT-qPCR (Figure 3). Mammalian cells exposed to high levels of LA show increased NFκB transcriptional activation and heightened levels of oxidative stress markers (Toborek et al., 1996; Poletto et al., 2015). Indeed, LA has been proposed to be detrimental to the health of hibernators for its capacity to undergo autooxidation and create lipid peroxides that can invoke inflammatory signaling, especially in hibernator adipose (Frank and Storey, 1995). The activity of antioxidant enzymes including SOD2 was increased in the brown adipose tissue of golden-mantled ground squirrels fed a high LA diet (Frank and Storey, 1995). More generally, naturally hibernating ground squirrels also increase the protein levels of antioxidants including superoxide dismutase 1 (SOD1), SOD2, and thioredoxin 1 (TRX1) as a potential mechanism to prevent ROS-mediated tissue damage in white adipose tissue (Rouble et al., 2014). Thus, an increase in the transcript levels of *sod2* in torpid dormice fed a diet high in LA relative to torpid low LA dormice are in line with previous findings. These results suggest the existence of a relationship between high LA diet in torpid dormice and antioxidant expression, suggesting increased oxidative stress in WAT and perhaps, increased NFκB activity.

Like the expression of *sod2, bax*, and *bclxl*, the levels of proteins downstream of NFκB (total c-Myc protein and phosphorylated FADD at Ser194 were measured to help determine if the activity of NFκB was indeed influenced by diet or hibernation status and an interesting correlation was discovered (Figure 2). While there were no changes in the relative protein expression of c-Myc in dormice fed a high LA diet, p-FADD levels (Ser194) were observed to decrease in torpid animals compared to euthermic controls, in line with the aforementioned results that suggest NFκB signaling is inhibited in dormice fed a high LA diet. FADD is an adapter molecule that binds the Fas receptor and promotes the activation of pro-apoptotic caspases through the formation of the death-inducing signaling complex (DISC) (Elmore, 2007). FADD phosphorylation at the C-terminal Ser194 encourages non-apoptotic signaling such as cell cycle progression and nuclear translocation (Alappat et al., 2005). Importantly, FADD phosphorylation is highest in the G2/M phases and lowest in the G1/S phases of the cell cycle (Alappat et al., 2005). Consistent with data from the hibernating 13-lined ground squirrel liver showing overall cell cycle arrest during torpor (Wu and Storey, 2012), decreased p-FADD S194 levels in dormouse WAT during torpor suggest overall cell cycle inhibition. Furthermore, gene expression levels of pro-apoptotic *bax* were not significantly increased in torpid dormice fed a high LA diet relative to torpid dormice fed a low LA diet. Together, these results suggest that animals fed a high LA diet may have reduced NFκB signaling, mediated by changes in phosphorylation of NFκB regulators (IKKα/β and IκBα), that manifest in torpid garden dormice as reductions in p-FADD levels.

In hibernating dormice fed low LA diet, there was significantly less *sod2* mRNA and c-Myc protein compared to the euthermic control group fed the low LA diet (Figures 2, 3). Additionally, c-Myc levels were significantly lower in dormice fed a low LA diet compared to torpid dormice fed an intermediate amount of LA. The similar trends in *sod2* and c-Myc suggest that the ratios of *n*−3 and *n*−6 PUFAs play an important role in transcriptional control in hibernating dormice. More specifically, analysis of the multiplex and RT-qPCR datasets suggested that diets deficient in LA, the fatty acid reported to be important for successful hibernation, could decrease NFκB-mediated gene expression in dormouse WAT during torpor. This was expected because diets high in LA have been suggested to impart higher NFκB binding activity, and because the low LA diet is high in ALA, an omega-3 fatty acid that can bind to PPAR to inhibit NFκB activity (Nakanishi and Tsukamoto, 2015; Poletto et al., 2015). Interestingly, previous studies suggest that c-Myc levels fall before the onset of apoptosis (Wood et al., 1994), so dormice fed a diet low in LA may be at increased risk of white adipose cell death during torpor, compared to dormice fed a diet with intermediate LA or high LA concentrations. Oppositely, hibernating dormice fed a high LA diet are likely less susceptible to cell death since p-FADD decreased during torpor compared to euthermic dormice who were fed a high LA diet. These results were consistent with the decrease in p-IKKα/β (Ser177/Ser181) levels in dormice fed a high LA diet. Overall, NFκB signaling seemed to be inhibited in WAT from dormice fed diets with high or low levels of LA, with correlated differences in the NFκB downstream proteins affected by such changes in diet.

### BAT Shows Opposite Trends in NFκB Signaling When Dormice Are Fed Diets High and Low in LA

**Figure 4:**
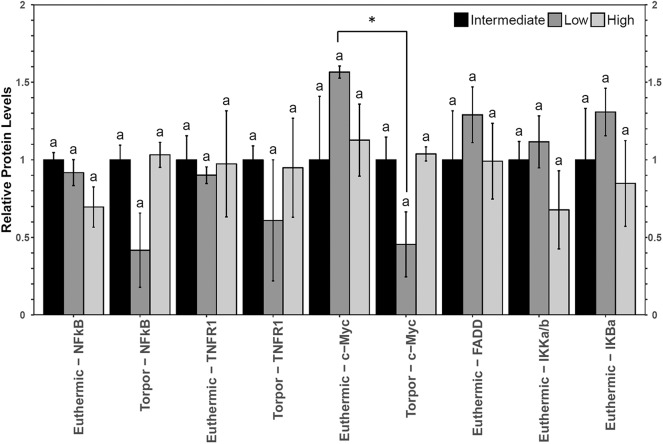
Multiplex analysis of key NFκB proteins in garden dormouse brown adipose tissue (BAT) including phosphoproteins [p-NFκB (Ser536), p-FADD (Ser194), p-IKKα/β (Ser177/Ser181), and p-IκBα (Ser32)] and total protein (c-myc and TNFR1). Mean expression levels are shown (±SEM, *n* = 3–4 independent protein isolations from different animals). Other information as in Figure 2.

**Figure 5:**
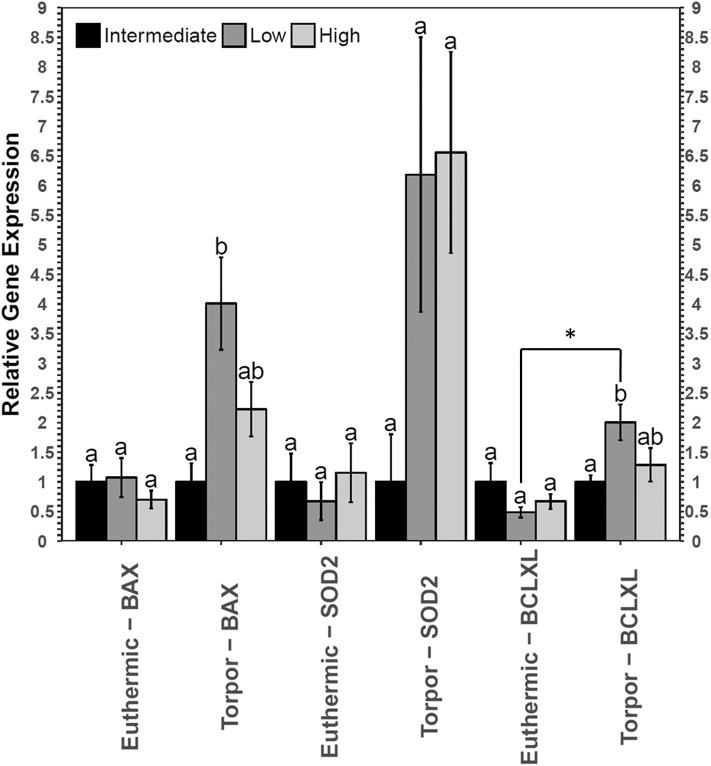
Relative gene expression levels of NFκB target genes, bax, sod2, and bclxl in garden dormouse brown adipose tissue (BAT). Histogram shows mean relative cDNA levels following normalization with the reference gene, ppia (±SEM, *n* = 4 independent protein isolations from different animals used for all genes except *n* = 3 used for bclxl quantification from euthermic dormice in the low LA condition). Diet data were analyzed using a One-Way ANOVA followed by a Tukey *post-hoc* test, where shared letters represent no significant difference between those diets (*p* > 0.05) and different letters represent significant differences between the diets (*p* < 0.05). A Student's *t*-test was used to determine if there were any significant differences in transcript levels comparing euthermic controls and hibernated animal groups. Asterisks (*) above the bar that designates the compared points indicates statistical significance with respect to the euthermic control time point.

Both diet and torpor were correlated with the expression of NFκB proteins and/or phosphorylation levels in brown adipose tissue (BAT), a tissue responsible for tight thermoregulation in heterothermic mammals. Similar to WAT, relative TNFR1 and p-NFκB (Ser536) levels did not change in BAT with hibernation status or diet (Figure 4). Unfortunately, BAT protein samples from animals fed low LA diets that were sacrificed during torpor had very low p-FADD, p-IKKα/β and p-IκBα signal relative to the no-protein control, so it was not possible to see the effects of diet on the expression of these proteins during torpor. Since a low LA diet generally decreases the expression of proteins downstream of NFκB, it was anticipated that gene expression of genes downstream of NFκB would also be inhibited. As expected, and similar to the expression profile in WAT, c-Myc protein levels decreased during torpor compared to euthermia, reinforcing the notion that a low LA diet may decrease NFκB activity. Decreased c-Myc levels in torpid dormice fed a low LA diet could indicate that cell cycle progression is decreased, and/or that BAT cells are being primed for apoptosis (Thompson, 1998). In line with the latter idea, the low LA diet increased pro- and anti-apoptotic gene expression (i.e., *bax* and *bclxl*, respectively) relative to an intermediate LA diet during torpor (Figure 5). Increased anti-apoptotic protein expression is also reported in other animals capable of metabolic suppression and likely promotes cell viability in response to environmental stress (Rouble et al., 2013; Gerber et al., 2016). Changes in gene expression such as those observed in dormouse BAT may be used to maintain metabolic homeostasis in animals fed unconventional ratios of *n*−3 and *n*−6 PUFA diets in advance of torpor. Obligate hibernators require a diet with LA levels that are not too high or too low. For instance, ground squirrels housed in laboratory environments hibernate best if they are given diets with LA levels that resemble those they would receive in the wild (i.e., higher than standard laboratory chow diets) (Frank and Storey, 1995). Indeed, in addition to ground squirrels, chipmunks, marmots, deer mice, and gliders all need diets with high levels of LA to hibernate optimally, but increasing LA levels above those experienced in the wild may not be beneficial based on relatively unaltered torpor patterns (Frank and Storey, 1995).

Instead, increasing the ratio of LA to ALA in the diet may increase pro-inflammatory NFκB signaling in the brown adipose tissue of dormice. Multiplex results showed that BAT from euthermic dormice fed a high LA diet had decreased p-IκBα (Ser32) levels relative to dormice fed low LA or intermediate LA diets. Low p-IκBα levels could be indicative of phosphorylated IκBα protein, its ubiquitination and subsequent degradation, so these results suggest that high LA diets could increase NFκB signaling. However, low p-IκB levels could also imply an inhibition of NFκB signaling through the maintained interaction between IκB and NFκB. An increase in NFκB signaling would be consistent with the literature, where diets high in LA lead to increased NFκB activity through the phosphorylation of IKKα/β (Poletto et al., 2015). However, the levels of downstream genes converted into protein (c-myc and FADD), as well as *bax, bclxl* or *sod2* were maintained in dormice fed a high LA diet, suggesting NFκB may have roles in BAT outside of mediating apoptosis and antioxidant defenses, such as upregulating pro-inflammatory adipocytokines. Of note, immunohistochemistry studies are required to confirm the presence of NFκB in the nucleus, and ChIP-sequencing studies would produce additional insights on how changes in the ratio of *n*−3/*n*−6 PUFAs in the pre-hibernation diet influence transcriptional regulation by NFκB.

### Liver Uses Antioxidant Defenses in Dormice Fed a High LA Diet but May Be Susceptible to Apoptosis When Fed a Diet High in *n*−3 PUFAs

**Figure 6:**
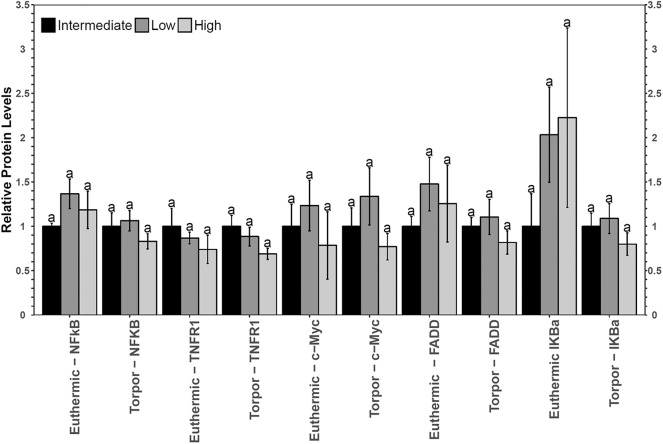
Multiplex analysis of key NFκB proteins including phosphoproteins [p-NFκB (Ser536), p-FADD (Ser194), p-IKKα/β (Ser177/Ser181), and p-IκBα (Ser32)] and total protein (c-myc and TNFR1) in liver from garden dormouse fed a diet intermediate, low, or high in LA. Mean expression levels are shown (±SEM, *n* = 4 independent protein isolations from different animals). Other information as in Figure 2.

**Figure 7:**
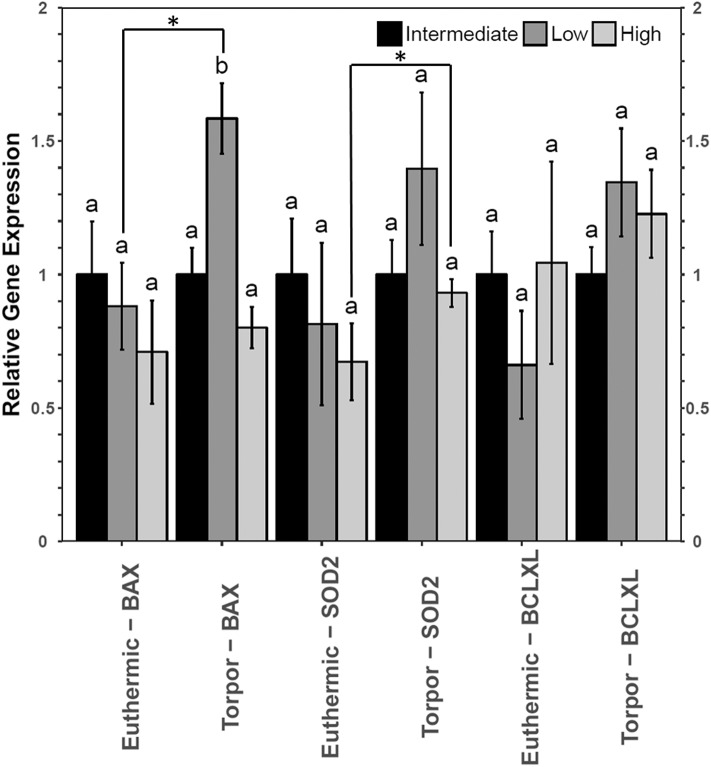
Relative gene expression levels of NFκB target genes, bax, sod2, and bclxl in garden dormouse liver. Histogram shows mean relative cDNA levels following normalization with the reference gene, ppia (±SEM, *n* = 4 independent protein isolations from different animals). Other information as in Figure 3.

Relative total protein and phosphorylation levels of the NFκB signaling pathway proteins did not change in liver of dormice fed any of the three diets (Figure 6), suggesting that these levels of dietary LA and ALA might not be enough to invoke an observable change in liver or perhaps the liver has sufficient mechanisms to detoxify lipid-derived ROS that have been proposed to increase NFκB transcriptional activity in hibernating animals (Frank and Storey, 1995; Carey et al., 2000). Interestingly, liver was the only organ to increase *sod2* gene expression during torpor relative to euthermia (Figure 7). Specifically, antioxidant gene expression only increased in the dormice fed a high LA diet, which is consistent with reports of increased lipid peroxidation with high LA feeding (Frank and Storey, 1995). Further, upregulation of antioxidant expression in liver makes intuitive sense, since liver is the major site of β-oxidation of fatty acids in torpid animals (Andrews, 2004; Williams et al., 2005). Similarly, the warm-torpid gray mouse lemur increases total antioxidant capacity in liver but not in any of the other 5 tissues relative to the non-hibernating control (Wu et al., 2015). Indeed, LA is extremely likely to undergo autooxidation and for this reason, it is unsurprising that compared to other fatty acids, LA is able to increase oxidative stress and TNF-α-mediated NFκB signaling following relatively brief exposures to this fatty acid (Toborek et al., 1996). High LA diets (relative to low LA diets) can increase mitochondrial disruption in hepatocytes, observed as decreased ATP and mitochondrial complex 1 protein levels (Schuster et al., 2018). Additionally, high LA diets can increase both oxidative stress and inflammation, measured as increased TXNIP and pro-IL-1β protein levels (Schuster et al., 2018). Importantly, *sod2* expression is highly increased by NFκB in response to both a shift in redox status (i.e., changes in ROS levels) but also in response to changes in the levels of pro-inflammatory cytokines such as TNFα, IL-6, IL-1β, and others, which may increase in the cell due to ROS-mediated tissue damage (Visner et al., 1990; Dougall and Nick, 1991; Xu et al., 1999). Antioxidant levels (vitamin E) increase in mammalian cells exposed to LA (Toborek et al., 1996), so an increase in *sod2* could be invaluable for high LA-fed dormice attempting to maintain homeostasis during torpor. Thus, the increased expression of *sod2* during torpor in dormice fed a high LA diet could indicate an attempt to detoxify hepatic tissue of LA-derived reactive oxygen species.

Despite no changes in upstream level of NFκB signaling proteins, dormice fed a diet low in LA diet increased *bax* gene expression relative to the euthermic control group fed the same diet, and relative to other torpid dormouse groups fed either of the other two diets. This is interesting because in combination with what we observe in WAT and BAT, these results suggest that a diet low in LA could increase apoptosis in all dormice tissues. Studies have shown that diets high in *n*−3 PUFAs may prevent hibernation in yellow-bellied marmots and in garden dormice (Hill and Florant, 2000; Giroud et al., 2018). The results of the current study suggest a low LA diet may increase cell death in multiple cell types, potentially prompting an early arousal from torpor to remove cellular debris in euthermic conditions.

## Conclusion

Together, the results of this study suggest that changes to the *n*−6/n-3 PUFA ratio has an impact on NFκB signaling in dormice, and especially during hibernation. High LA diets were anticipated to increase protein and mRNA biomarkers of NFκB signaling as well as relative phosphorylation levels, since LA can alter the phosphorylation status of NFκB regulators IKKα/β and IκB, and LA metabolites like arachidonic acid can increase the production of pro-inflammatory eicosanoids and cytokines that increase NFκB signaling (Schmitz and Ecker, 2008; Poletto et al., 2015). However, high LA diets were correlated with a decrease in p-IKKα/β (relative to other diets) and p-FADD (relative to euthermia), suggesting NFκB signaling is decreased in WAT. High LA diets can induce the expression of other transcription factors such as AP1 that are capable of increasing *sod2* gene expression (Miao and St. Clair, 2009; Yu et al., 2017). Therefore, future studies should focus transcription factors other than NFκB to help explain the increase in sod2 expression in WAT from dormice fed a high LA diet. Consistently, euthermic dormouse BAT decreased p-IκB relative to other diets, suggesting NFκB signaling could be enhanced by a high LA diet but only maintained downstream biomarkers supported this. Supported by no increases in p-IKKα/β levels in dormice fed the high LA diet, an alternative explanation could include an inhibition of NFκB signaling such that total IκB protein levels are maintained and phosphorylation is decreased. An increase in *sod2* expression with high LA diet in liver was interesting because β-oxidation may increase with higher levels of *n*−6 PUFAs during torpor. In comparison to humans, hibernators fed high levels of LA may be more adept at preventing inflammation e.g., via NFκB signaling in their tissues. By contrast, a low LA diet high in *n*−3 PUFAs may contribute excess cellular stress since decreased c-Myc levels, indicative of cell senescence and apoptosis, were observed in both types of adipose tissue. Further, BAT and liver increased the levels of apoptotic mediators *bax* and *bclxl* with changes in diet and/or during torpor. Notably the low LA diet invoked an increase in *bax* gene expression during torpor that was not matched by a change in *bclxl* expression, suggesting an imbalance in apoptosis mediators favoring cell death in liver cells from dormice fed low levels of *n*−6 PUFA. Taken together, the results support the hypothesis that a change in PUFA composition influences inflammatory gene expression in hibernating dormice, and these changes are tissue-specific. The results of this study further extend our understanding of how human metabolism differs from that of a species capable of metabolic depression and provides reason for carefully controlling the *n*−6/n-3 PUFA content of diets used in hibernation experiments on breeding colonies.

## Data Availability Statement

All datasets generated for this study are included in the article/supplementary files. PCR and Multiplex data are available at Figshare: Logan, Samantha (2019) Dormouse NFκB PCR data. figshare. Dataset (https://doi.org/10.6084/m9.figshare.12280634) and Logan, Samantha (2019): Dormouse Luminex data. figshare. Dataset (https://doi.org/10.6084/m9.figshare.12280652).

## Ethics Statement

The animal study was reviewed and approved by the institutional ethics committee and the national Austrian authority in accordance to the Austrian Animal Experimentation Act, Tierversuchsgesetz 2012 (BMBWF-68.205/0137-WF/V/3b/2014).

## Author Contributions

Conceptualization and methodology of the molecular analyses was conducted by SL, AW, and KS. Methodology (model creation, animal setup, and experiments) was performed by AP, AK-H, and SG. JP and GS performed the animal surgeries. SL and AW completed the investigation and validation experiments. Formal analysis was conducted by SL and SG. Visualization and original draft preparation were performed by SL. Review and editing of the manuscript was done by SL, AW, SG, and KS. Resources and supervision were provided by SG and KS, who were also responsible for funding acquisition. KS performed project administration. All authors commented and agreed on the final version of the manuscript and participated in the revisions.

## Conflict of Interest

The authors declare that the research was conducted in the absence of any commercial or financial relationships that could be construed as a potential conflict of interest.

## Abbreviations

AA: Arachidonic acid
ALA: Linolenic acid
BAX: BCL2 Associated X Protein
BCLXL: BCL2 Like 1
C-myc: , Cellular MYC proto-oncogene, bHLH transcription factor
DHA: Docosahexaenoic acid
DISC: Death-inducing signaling complex
EPA: Eicosapentaenoic acid
FADD: Fas Associated Via Death Domain
GLUT4: Glucose Transporter 4
IκBα: NF-Kappa-B Inhibitor Alpha
IKKα/β: Inhibitor Of Nuclear Factor Kappa B Kinase subunits alpha and beta
IL: Interleukin
LA: Linoleic acid
NFκB: Nuclear Factor Kappa B
PPAR: Peroxisome Proliferator Activated Receptor
PUFA: Polyunsaturated fatty acid
SOD2: Superoxide Dismutase 2
TNFα: Tumor Necrosis Factor Alpha
TNFR1: Tumor Necrosis Factor Receptor 1
TXNIP: Thioredoxin Interacting Protein.
